# New Variants of the Cytochrome P450 2R1 (*CYP2R1*) Gene in Individuals with Severe Vitamin D-Activating Enzyme 25(OH)D Deficiency

**DOI:** 10.3390/biom11121867

**Published:** 2021-12-12

**Authors:** Martyna Fronczek, Joanna Katarzyna Strzelczyk, Krzysztof Biernacki, Silvia Salatino, Tadeusz Osadnik, Zofia Ostrowska

**Affiliations:** 1 Department of Medical and Molecular Biology, Faculty of Medical Sciences in Zabrze, Medical University of Silesia, 40-055 Katowice, Poland; jstrzelczyk@sum.edu.pl (J.K.S.); kbiernacki@sum.edu.pl (K.B.); ozdrasiek@wp.pl (Z.O.); 2 Department of Pharmacology, Faculty of Medical Sciences in Zabrze, Medical University of Silesia, 40-055 Katowice, Poland; tadeusz.osadnik@sum.edu.pl; 3 Molecular Biology, Core Research Laboratories, Natural History Museum, London SW7 5BD, UK; s.salatino@nhm.ac.uk

**Keywords:** vitamin D, metabolism of vitamin D or its analogs, cytochrome P450, *CYP2R1*, 25-hydroxylase

## Abstract

Background: Vitamin D is a fat-soluble cholesterol derivative found in two forms, vitamin D2, and vitamin D3. Cytochrome P450 2R1 (CYP2R1) encoded by the *CYP2R1* gene is the major hydroxylase that activates vitamin D by catalyzing the formation of 25-hydroxyvitamin D (25(OH)D). Methods: We collected 89 (100%) subjects, 46 of which (51.69%) had a documented severe deficiency of 25(OH)D (<10 ng/mL) and 43 (48.31%) in the control group with documented optimum levels of 25(OH)D (>30 ng/mL). We performed Sanger sequencing of three selected fragments of the *CYP2R1* gene (Ch11: 14878000–14878499; Ch11: 14880058–14880883 and Ch11: 14885321–14886113) that affect the binding of substrates to this enzyme and analyzed the possible involvement of genetic variation in these regions with an increased risk of vitamin D deficiency in healthy Polish individuals. Results: Two substitutions were found within the three fragments. Bioinformatic analysis suggested that one of these (NC_000011.10: g.14878291G>A) may influence the structure and function of CYP2R1. Conclusions: Variant NC_000011.10: g.14878291G>A may have a perturbing effect on heme binding in the active site of CYP2R1 and on the function of 25-hydroxylase and probably affects the concentration of 25(OH)D in vivo. We intend to perform functional verification in a larger patient population to confirm and extend these results.

## 1. Introduction

Vitamin D is a fat-soluble cholesterol derivative found in two forms that differ in their side-chain structure: vitamin D2 (ergocalciferol, naturally occurring in plant organisms) and vitamin D3 (cholecalciferol, produced in animal organisms) [1,2]. There are two main sources of vitamin D: synthesis in the skin from cholesterol and food products (diet and vitamin supplements) [3,4]. Although initial studies on vitamin D mainly focused on bone metabolism, this molecule has recently become the subject of interest in other fields of medicine and has been suggested to play a key role as a risk factor for cardiological diseases (CVD) including stroke, atherosclerosis, myocardial infarction (MI), and coronary artery disease (CAD) [5,6,7]. At present, according to the guidelines the optimal concentration of 25-hydroxyvitamin D (25(OH)D) in serum to ensure the pleiotropic effect of this vitamin is from 30 to 50 ng/mL (75–125 nmol/L) [8]. A concentration of 20–30 ng/mL (50–75 nmol/L) is considered suboptimal; values in the range 10–20 ng/mL (25–50 nmol/L) are defined as moderate deficiency, while those below 10 ng/mL (below 25 nmol/L) are considered as severe vitamin D deficiency. A serum 25(OH)D concentration above 100 ng/mL (above 250 nmol/L) can be toxic for the human body [8,9].

Cytochrome P450 2R1 (cytochrome P450 family 2 subfamily R member 1, CYP2R1) encoded by the *CYP2R1* gene located at the p15.2 locus of chromosome 11, is the major hydroxylase that converts vitamin D in the liver to the intermediate 25(OH)D [10,11]. The *CYP2R1* gene contains five exons that are translated into a 501-amino acid protein with a molecular weight of 57,359 Da, which has the structure of a typical cytochrome P450 [12]. The structure of CYP2R1 is stabilized by electrostatic interactions between the C-terminal end of the G-helix of one molecule (Arg259 and Asp255) and the N-terminal residues of the F-helix of the other molecule (Asp206 and His209). It consists of α (A–L) helices, β sheets, and heme located deep inside the protein. The E, I, J, K, and L helices form the core of the enzyme, while the F and G helices are involved in creating its active center. The B′ helix is one of the substrate recognition sites (SRS). The active site is formed by 19 amino acid residues: Leu114, Phe115, Met118, Leu125, Asn126, Phe214, Asn217, Ala221, Ala250, Val253, Tyr254, Phe302, Glu306, Ala310, Thr314, Val375, Ile379, Met487, and Thr488 [13].

The promoter region of the *CYP2R1* gene is located within the CpG island, which may be epigenetically regulated. Fetahu et al. indicate increased methylation of the promoter in DNA of leukocytes from people with severe vitamin D deficiency [14]. In addition, the methylation level of the *CYP2R1* promoter was found to be reduced in postmenopausal women within 12 months of starting supplementation with vitamin D. This promoter may undergo methylation at low vitamin D concentrations in the serum, which is reversed after supplementation with this vitamin [14].

Genome-Wide Association Studies (GWAS) have provided valuable information on the role of *CYP2R1*. Variants within the *CYP2R1* gene have been identified that are potentially related to serum 25(OH)D concentration [15,16]. Previous studies focusing on the genetic, dietetic, and environmental aspects of 25(OH)D production [3,10,17], together with the high level of conservation of *CYP2R1* across all mammalian species, suggest a significant biological role of this molecule in ensuring vitamin D homeostasis in liver cells and in other cells of the body [18,19]. Understanding how genetic changes in the *CYP2R1* gene can affect the activity of this enzyme is a key to determining how these may predispose patients to diseases such as metabolic syndrome or CAD.

The aim of this study was to sequence three selected fragments of the *CYP2R1* gene, corresponding to the substrate binding and metal-binding sites of CYP2R1, to investigate the contribution of genetic variations to increased risk of vitamin D deficiency.

## 2. Materials and Methods

### 2.1. Patients

We collected 89 (100%) healthy subjects between 18 and 35 years of age, 46 of which (51.69%) had a documented severe vitamin D deficiency (defined as serum 25(OH)D <10 ng/mL) and were used as a study group, while the remaining 43 (48.31%) had a documented optimum level of vitamin D (defined as >30 ng/mL) and were used as a control group. The study group included 25 (54.35%) men and 21 (45.65%) women, whereas the control group consisted of 21 (48.84%) men and 22 (51.16%) women. Subjects were recruited from the Silesian Centre for Heart Diseases in Zabrze, Poland between July 2015 and October 2017 as part of the MAGNETIC (Metabolic and Genetic Profiling of Young Adults with and without a Family History of Premature Coronary Heart Disease) Study. Patients’ anthropometric, biochemical, lifestyle-related results, and values of Intima-Media Thickness (IMT) and Visceral Adiposity Index (VAI) were obtained from the MAGNETIC project [20]. The criteria for inclusion in the study group were: 25(OH)D concentration below 10 ng/mL, age over 18 and under 35, no chronic diseases requiring pharmacotherapy, and no vitamin supplementation. For the control group, the criteria were identical except for 25(OH)D concentration, which was required to be above 30 ng/mL. Exclusion criteria included: pregnancy, lactation, and the lack of signed informed consent for the study.

### 2.2. Biochemical Analyses

Peripheral blood was collected from each patient to test selected biochemical and immunochemical parameters (cholesterol, LDL, HDL, TG, Lp(a), Apo A, Apo B, hsCRP, FG, HBA1C, bilirubin, ALAT, ASPAT, GGT, fibrinogen, ALP, 25(OH)D, and total calcium, phosphorus). Blood was drawn during the morning recruitment visit and on an empty stomach (8–10 h after the last meal) into three different tubes: S-Monovette tubes coated with EDTA (Sarstedt AG&Co. KG., Nümbrecht, Germany) for whole blood or plasma, S-Monovette tubes with a clotting activator (Sarstedt AG&Co. KG., Nümbrecht, Germany) for serum, and S-Monovette tubes with 3.1% sodium citrate (Sarstedt AG&Co. KG., Nümbrecht, Germany) for fibrinogen content. The collected blood was centrifuged at 1500 rpm for 10 min at 4 °C to obtain serum. Measurements were made using a Cobas 6000 analyzer (Roche Diagnostics, Indianapolis, IN, USA) at 3000 rpm for 10 min at 4 °C for plasma. The fibrinogen content was determined using a BCS XP analyzer (Siemens Healthcare, Erlangen, Germany).

### 2.3. CYP2R1 Gene Sequencing

#### 2.3.1. DNA Isolation

DNA was isolated using the MagCore^®^ Genomic DNA Whole Blood Kit (RBC Bioscience Corp., New Taipei City, Taiwan) based on separation on magnetic beads and quantitated using an Epoch™ spectrophotometer (BioTek Instruments, Inc., Winooski, VT, USA).

#### 2.3.2. PCR of Selected Fragments of the CYP2R1 Gene

Three fragments of the *CYP2R1* gene were selected for Sanger sequencing analysis using the Primer-BLAST software (https://www.ncbi.nlm.nih.gov/tools/primer-blast/index.cgi?LINK_LOC=BlastHome, accessed on 9 December 2021) available from the National Center of Biotechnology Information (NCBI). Primers were synthesized and purified by HPLC (Genomed, Warsaw, Poland). Primer sequences are provided in Supplementary Table S1.

PCR reactions for selected fragments of the *CYP2R1* gene were carried out using DyNAzyme II DNA Polymerase (Thermo Fisher Scientific, Waltham, MA, USA) in a SimpliAmp™ Thermal Cycler (Thermo Fisher Scientific, Waltham, MA, USA). The conditions for each pair of primers were determined experimentally (Supplementary Tables S2 and S3).

#### 2.3.3. Purification of the CYP2R1 PCR Products

Each PCR product was incubated with Exo-BAP Mix (Eurx, Gdańsk, Poland) to remove primers and free nucleotides by thermo-sensitive alkaline phosphatase and Exonuclease I. The reaction was carried out using the manufacturer’s recommendations (Supplementary Table S4).

#### 2.3.4. Fluorescence-Based Cycle Sequencing Reaction

After purification of PCR products, their concentration was measured by a QuantiFluor dsDNA System (Promega Corporation, Madison, WI, USA) using a Quantus Nucleic Acid Quantification Fluorometer (Promega Corporation, Madison, WI, USA) according to the manufacturer’s instructions. To perform a cycle sequencing reaction, purified PCR products were used to assemble the reaction using the reagents of the dedicated ABI PRISM^®^ BigDye^®^ Terminator v3.1 Cycle Sequencing Kit (Thermo Fisher Scientific, Waltham, MA, USA) according to the instructions of the manufacturer (Supplementary Table S5).

#### 2.3.5. Purification of Templates after Cycle Sequencing Reaction

Samples were kept on a microplate vortex mixer for 30 min at 2000 rpm at room temperature (IKA MS3 Digital, IKA Werke GmbH&Co. KG, Staufen, Germany) with BigDye^®^ XTerminator^TM^ Purification Kit solutions (Thermo Fisher Scientific, Waltham, MA, USA) to remove salts and unincorporated BigDye^®^ terminators (Supplementary Table S6). Next, samples were centrifuged at 1890 rpm for 2 min in a swinging-bucket centrifuge, and the supernatant was used for separation in the analyzer.

#### 2.3.6. Capillary Electrophoresis

Separation of the purified PCR products was carried out in a 3130 Genetic Analyzer (Applied Biosystems, Foster City, CA, USA) using a 3130/3100-Avant 4-Capillary Array, 36 cm (Thermo Fisher Scientific, Waltham, MA, USA) designed for sequencing and fragment analysis. The 3130 POP-7TM Performance-Optimized Polymer (Thermo Fisher Scientific, Waltham, MA, USA) and dedicated by manufacturer buffer with EDTA (Thermo Fisher Scientific, Waltham, MA, USA) were used for sample separation. The results of the analysis were collected using the 3130 Genetic Analyzer software (Applied Biosystems, Foster City, CA, USA).

### 2.4. Bioinformatic Analysis

Trace files (in AB1 format) were inspected using the Applied Biosystems Sequencing Analysis Software (Thermo Fisher Scientific, Waltham, MA, USA). Bioinformatic data analysis was carried out by the Molecular Biology Core Research Laboratory of the Natural History Museum in London, using in-house developed Python scripts in conjunction with Tracy [21] for trace decomposition of forward and reverse strands, BCFtools [22] for InDels left alignment and merging of the normalized variants across the three fragments of the *CYP2R1* gene, and VEP [23] for variant annotation to the human GRCh38 reference genome. Annotation databases included HGVS nomenclature, allele frequencies from large genomic projects (1000 Genomes, ExAC, GnomAD), as well as Ensembl transcript identifiers and mutation consequences on the downstream protein sequence. The detected variants were confirmed using GeneStudio^TM^ Pro 2.2.0.0 software (GeneStudio Inc., Suwanee, GA, USA) [24].

Three different prediction tools were used to verify the effect of the observed substitutions in *CYP2R1*: SIFT, PolyPhen, and SpliceAI. SIFT predicts the probability of amino acid substitution effects on protein function based on sequence homology and physicochemical similarity between alternative amino acids. Variants with scores in the range of 0.0 to 0.05 are considered deleterious, whereas variants with scores in the range of 0.05 to 1.0 are predicted to be tolerated (benign) [25,26]. PolyPhen-2 predicts the effect of amino acid substitution on protein structure and function using sequence homology and databases such as Pfam or 3D structures from PDB. The score ranges from 0.0 (tolerated) to 1.0 (deleterious), and values closer to 1.0 are more confidently predicted to be deleterious. The score can be interpreted as benign (test value ≤0.446); possibly damaging (test value >0.446 and ≤0.908), or probably damaging (test value >0.908) [26,27]. SpliceAI is a residual convolutional neural network by which the probability of RNA splicing in donor or acceptor DNA sequences can be predicted. SpliceAI calculates four delta scores (DS) for each variant, indicating the likelihood of a given variant to result in acceptor gain (AG), acceptor loss (AL), donor gain (DG), or donor loss (DL) [28,29]. Delta scores range from 0 to 1 and can be interpreted as the probability of the variant being splice-altering. *CYP2R1* variants were analyzed by SpliceAI to predict the types of RNA splicing resulting from genetic variation. We calculated the probability of RNA splicing types for alternative nucleotides based on reference sequences (GRCh38/hg38). Each DS score is defined as the maximum difference between the reference and alternative probabilities at the splicing sites. SpliceAI also provided the position of acceptor/donor loss or gain, which indicates the location of splicing changes with probability relative to the position of interest; positive delta position values indicate that splicing changes are downstream of the variant, while negative values are upstream [29].

### 2.5. Statistical Methods

The STATISTICA v.13.3 software (StatSoft, Kraków, Poland) was used for statistical analysis of the results. Clinical data of the study groups are presented in the form of the median and interquartile range, while the data in the nominal scale are presented as a percentage of the total. The comparison of clinical data between the control group and the study group was carried out using the Mann–Whitney U test and the Pearson chi-square test. A significance level of *p* < 0.05 was adopted for all statistical analyses.

## 3. Results

Bioinformatic analysis of the sequencing results was carried out in 46 subjects with a concentration of 25(OH)D below 10 ng/mL (mean concentration 7.13 ng/mL ± 2.16 ng/mL) and in 43 subjects with the normal concentration of 25(OH)D (mean concentration 43.40 ng/mL ± 8.63 ng/mL). Biochemical and anthropometric parameters, as well as data related to lifestyle, are presented in Table 1.

**Figure 1:**
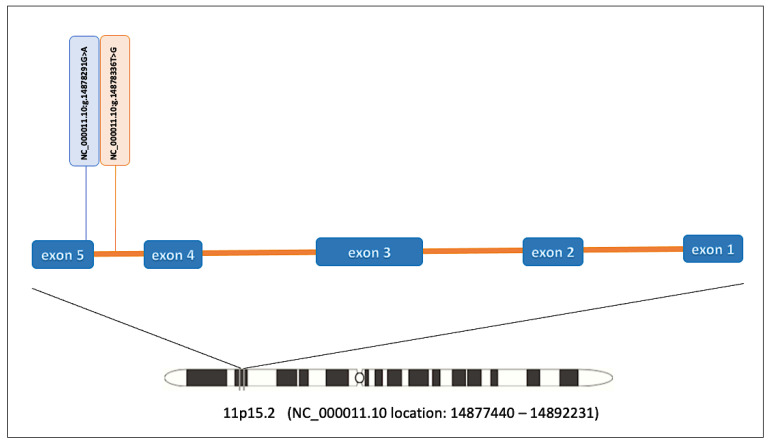
Structure of the human cytochrome P450 family 2 subfamily R member 1 (*CYP2R1*) gene and designation of the NC_000011.10:g.14878291G>A and NC_000011.10: g.14878336T> G variants detected here. The location of the human gene is based on data obtained from Genome Data Viewer [30].

A total of 159 mutations were found in the 3 investigated fragments of the *CYP2R1* gene among the 89 participants. 44 mutations occurred in fragment 1, 30 in fragment 2, and 85 in fragment 3. After evaluating these variants with GeneStudio^TM^ Pro 2.2.0.0. software (GeneStudio Inc., Suwanee, GA, USA), the number of detected changes was narrowed down to only two substitutions (Figure 1). These occurred in 10 patients, with 1 variant being found in 9 patients and both variants in 1 patient. Eight of the 10 participants who had 1 mutation were from the group with vitamin D deficiency, and the remaining 2 were from the control group.

**Figure 2:**
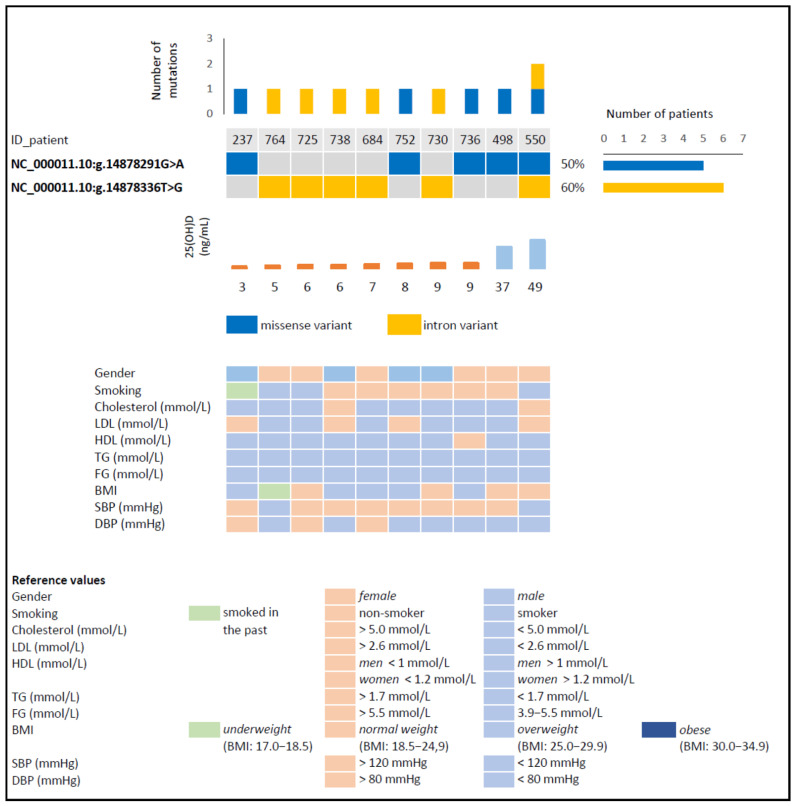
Basic characterization of patients with detected variants of the selected fragments of the *CYP2R1* gene. Abbreviations: 25(OH)D—25 hydroxyvitamin D; LDL—low-density lipoprotein; HDL—high-density lipoprotein; TG—triglycerides; FG—fasting glucose; BMI—body mass index; SBP—systolic blood pressure; DBP—diastolic blood pressure.

The predicted effect of these amino acid substitutions on the structure and function of CYP2R1 was analyzed using SIFT and PolyPhen prediction tools. Additionally, the potential for acquiring or losing alternative splicing sites was verified by SpliceAI. The first detected variant, NC_000011.10:g.14878291G>A (c.1337G>A), is a missense mutation that probably impairs (or damages) the structure and function of the protein—the value of the PolyPhen score analysis was 1.0, and for SIFT, it was 0.0. This variant led to a replacement of arginine (Arg) with lysine (Lys) at codon 446 (p.Arg446Lys) and was detected in 3 subjects with a 25(OH)D concentration below 10 ng/mL and in 2 persons from the control group (Figure 2). The result of the SpliceAI analysis (DS score = 0.25 (6 bp)) suggests that this variant may have lost a splicing acceptor site (Table 2), and additionally, it should be noted that Arg446 is one of the heme-binding sites [31]. This variant has not been reported in individuals with CYP2R1-related or other conditions, nor in population databases (no frequency in ExAC); however, in the dbSNP database, another variant can be found at this position (rs1555010445->GT, ExAC 0.000%). Bioinformatic algorithms designed to predict the effect of missense changes on protein structure and function (SIFT, PolyPhen-2) suggest that this variant may be impairing; however, this prediction has not yet been confirmed in published functional studies.

The second detected variant (NC_000011.10: g.14878336T>G (c.1331-39T>G) is an intron variant and was identified in 5 subjects with a concentration of 25(OH)D below 10 ng/mL and in 1 person with a concentration above 30 ng/mL (Figure 2). SpliceAI analysis did not show any significant effect on alternative splicing (Table 2). This variant is not present in population databases (no frequency in ExAC) and has not been reported in the literature in individuals with CYP2R1-related or other conditions.

## 4. Discussion

The missense mutation described in our study at position NC_000011.10:g.14878291G>A of the *CYP2R1* gene results in an Arg substitution to Lys at position 446. Arg has a positively charged guanidine group and is often found at sites that bind to phosphorylated substrates [32]. Although six codons in the genetic code for Arg [33] this does not translate into a high frequency of this amino acid in proteins; indeed, arginine is one of the rarest amino acids found in proteins [32,34]. In turn, Lys is an amino acid whose positively charged ε-amino group is highly reactive and is often involved in reactions at the active centers of enzymes [35]. Studies of membrane proteins suggest that Arg and Lys may play a similar role [36]; both are basic with a high pKa value in water (12.0–13.7 for Arg and ~10.5 for Lys), but Arg provides greater stability to protein structure compared to Lys because its guanidinium group allows interaction in three possible directions through three asymmetric nitrogen atoms, while the Lys functional group allows only one direction of interaction [37]. This means that Arg has a greater potential to create more electrostatic interactions than Lys, and most likely, this contributes to the formation of stronger interactions; additionally, Arg produces more stable ionic interactions compared to Lys due to the higher pKa of its basic residue [38,39,40,41,42]. According to the literature, CYP2R1’s substrate selectivity may be explained by the interactions near the entrance to the substrate-binding site where sequestration of the secosterol portion of the substrate occurs. CYP2R1, similarly to other microsomal CYP proteins, uses both electrostatic interactions with the redox partner NADPH-cytochrome P450 reductase in complex formation. Mapping of interacting residues revealed their localization as Arg131, Arg137, and Arg145 in the C helix, Lys434, Lys435, and Arg445 in the meander region, and Arg455 in the L helix. Additionally, Trp133, Arg137, and Arg446 are involved in heme binding by hydrogen bonds to the D-ring propionate [13,31,43]. As shown by the results of SIFT, PolyPhen, and SpliceAI, the mutation identified may affect CYP2R1 protein function. Due to the fact that Arg446 is one of the residues involved in heme binding to the CYP2R1 active site, the detected substitution may affect its binding to CYP2R1 and may have a potential effect on substrate hydroxylation. This prediction has not yet been confirmed in published functional studies, so its clinical significance is uncertain and requires further verification and confirmation. The available evidence is insufficient to determine the role of this variant in 25(OH)D deficiency. The second intron substitution (NC_000011.10:g.14878336T>G) identified in this work has not been described in previous studies. Despite the lack of literature data, it can be concluded that these variants occurring in the *CYP2R1* gene may affect its structure and function.

Variants within the *CYP2R1* gene that are potentially associated with serum 25(OH)D concentration have been identified in other studies [15,16]. Published studies of the hereditary form of rickets caused by a defect in vitamin D 25-hydroxylation provide evidence for a significant role of this cytochrome as vitamin D 25-hydroxylase. The molecular basis for 25-hydroxylase deficiency in Cheng et al. [17] is an NC_000011.10:g.14885847A>G (p.Leu99Pro) mutation in exon 2 of the *CYP2R1* gene, and the role of Leu99 in CYP2R1 protein folding is consistent with the assumption that this mutation eliminates CYP2R1 activity. Additionally, analyses suggest that this mutant protein does not have vitamin D3 hydroxylation activity but does have residual vitamin D2 hydroxylation activity [17]. In addition, studies by Thacher et al. conducted among Nigerian children with familial rickets and vitamin D deficiency indicate a significant role of this and the c.726A>C (p.Lys242Asn) mutation in reducing 25-hydroxylase activity [12]. Other analyses of siblings with symptoms of vitamin D deficiency and rickets by al Mutair et al. detected two CYP2R1 mutations, NC_000011.10:g.14885775C>T (c.367+1G>A) and NC_000011.10:g.14880371dup (p.Leu257fs). The former mutation is within the highly conserved splicing sequence of intron 2 *CYP2R1* donor and may lead to the formation of an mRNA that is unstable or translated to a shorter protein, and the latter is a thymine insertion in exon 3 that leads to a frameshift mutation and to the formation of a shorter protein [44]. Another variant (NC_000011.10:g.14892069_14892082delinsCG (p.Gly42_Leu46delinsArg) described by Molin et al. may lead to the formation of a protein deficient in one of the four intramembrane hydrophobic domains, and in silico analyses indicate that it may lead to modification of the three-dimensional conformation of the substrate access channel and thus may disrupt the hydroxylation process [45].

Additionally, earlier studies indicate that selected polymorphisms of the *CYP2R1* gene are related to serum vitamin D concentration. The variant rs10741657 is located in the non-coding region of the 5′-UTR and can potentially modify the expression of the *CYP2R1* gene, which may translate into a change of enzymatic activity [46,47]. In a meta-analysis by Duan et al., the GG genotype was associated with a decreased concentration of 25(OH)D, and the G allele of this polymorphism was associated with an increased risk of vitamin D deficiency [47]. A similar trend has been reported in other studies [48,49,50]. Nissen et al. showed a carrier relationship of the G allele of this polymorphism with a lower concentration of vitamin D. Participants with a G allele (GG and GA) had a lower concentration of vitamin D compared to those with an AA genotype [50]. Lafi et al. showed higher concentrations of vitamin 25(OH)D in healthy participants with the A allele compared to the wild-type genotype of rs10741657 [48]. The relationship between the rs10741657 polymorphism and the concentration of 25(OH)D and CAD showed a significantly lower concentration of 25(OH)D in patients, and therefore this polymorphism has been proposed as a new genetic marker of CAD. The second variant analyzed in this study, rs12794714, did not show a relationship between its distribution and the concentration of 25(OH)D and CAD [51], confirming the meta-analysis by Duan et al. [47].

Vitamin D in the form of two analogs, D3 and D2, is an inactive compound that requires bioactivation for biological activity. Vitamin D3, synthesized from its precursor 7-dehydrocholesterol by exposure of the skin to UVB light, and vitamin D2 found in plants are first 25-hydroxylated mainly in the liver by CYP enzymes, for example, mitochondrial CYP27A1 (vitamin D3) or microsomal CYP2R1 (vitamin D3 and D2) [1,52]. In the next step, 25(OH)D is metabolized to the active metabolite, vitamin 1,25D (1,25(OH)D) by mitochondrial CYP27B1, which occurs mainly in the kidney. 1,25(OH)D interacts with the cell vitamin D receptor (VDR) and retinoid X receptor (RXR) and in this way acts as a transcriptional regulator of the expression of genes controlling proper cell growth, differentiation, repair, and apoptosis [1,10]. The results of the above studies suggest that CYP2R1 is the major vitamin D 25-hydroxylase, playing a fundamental role in its activation. Since CYP2R1 is highly conserved in all studied species of mammals and is present in many cells of the organism, it can be concluded that it plays an important role in vitamin D economy and in maintaining organism homeostasis [18]. The present results indicate that genetic changes within the sequence of the *CYP2R1* gene have significant effects on the process of vitamin D hydroxylation. The body’s supply of vitamin D also depends on lifestyle, as well as environmental and genetic factors. The synthesis of 25(OH)D depends on exposure to UV irradiation, age, skin pigment, and genetic changes [3,10]. CYP2R1 is considered to be the major vitamin D 25-hydroxylase, but it should be noted that the 25-hydroxylation process itself may also involve other CYP family enzymes, which also have 25-hydroxylase activity [53,54]. 25-hydroxylase activity has also been found in human mitochondrial CYP27A1 [55] or microsomal CYP3A4 [56] and CYP2J2 [57]. The literature shows that they differ in their substrate specificity in 25-hydroxylation of vitamin D3 and D2, catalytic properties, and tissues distribution [53]. Literature data indicate that the most critical 25-hydroxylase in humans is CYP2R1, as genetic variations in this enzyme, but not in CYP27A1, are responsible for serum 25(OH)D deficiency [17,58]. In addition, in a *CYP2R1* knockout mouse (*Cyp2r1^−^*^/*−*^ and *Cyp2r1^−^*^/*−*^/*Cyp27^−^*^/*−*^), a similar 50% reduction in 25(OH)D3 was found in both models. The remaining 50% was most likely provided by the functioning of other enzymes capable of 25-hydroxylating vitamin D3. Additionally, an increased *CYP2R1* transcript level was observed by real-time PCR in the *Cyp27a1^−^*^/*−*^ model. These observations indicate that CYP2R1 is the major but not the only 25-hydroxylase of vitamin D [59]. As there is evidence that CYP2R1 is the key vitamin D 25-hydroxylase [17,54,55], we believe that the mutations discovered here may have an impact on 25(OH)D formation. In the future, we plan to expand this analysis further by performing studies on *CYP2R1* and other CYP family enzymes expression and its vitamin D hydroxylation activity in human wild-type and mutant constructs, with a wider patient cohort [12,17,45]. The effect of the detected intron variant on splicing will be tested using splicing reporter minigene assay by transient cell lines transfection with a minigene construct [60,61].

## 5. Conclusions

The mutation NC_000011.10:g.14878291G>A in *CYP2R1* identified here may have a perturbing effect on heme binding in the active site of CYP2R1 and on the function of 25-hydroxylase and probably affects the concentration of 25(OH)D in vivo. Our conclusion should be verified by functional studies to confirm its clinical significance. Due to the lack of literature evidence on the effect of variants on vitamin D concentration, this functional validation should be conducted in a large patient population.

## Figures and Tables

**Table 1:** Comparison of anthropometric, biochemical and lifestyle data in the study and the control group.

Parameters	25(OH)D > 30 ng/mL *n* (%) 43 (100%)	25(OH)D < 10 ng/mL *n* (%) 46 (100)	*p*-Value U Mann–Whitney/^2^
Physical activity *low* *moderate* *high*	11 (25.58) 20 (46.51) 12 (27.91)	17 (36.96) 22 (47.82) 7 (15.22)	0.273
BMI (kg/m^2^)	23.42 (22.20; 25.15)	24.62 (22.43; 26.20)	0.440
FH of DM2	10 (23.26)	7 (15.22)	0.335
FH of P-CAD	27 (62.79)	29 (63.04)	0.980
WC (cm)	81.25 (75.00; 89.00)	86.00 (76.00; 92.00)	0.535
WHR	0.86 (0.82; 0.90)	0.85 (0.82; 0.89)	0.932
VAI	0.81 (0.65; 1.11)	1.12 (0.84; 1.33)	0.024
SBP (mmHg)	123.00 (120.00; 128.00)	126.71 (124.00; 135.00)	0.044
DBP (mmHg)	75.00 (72.00; 79.00)	81.50 (77.46; 90.00)	0.002
Smoking, *n* (%) *smoker* *non-smoker* *smoked in the past*	9 (20.93) 27 (62.79) 7 (16.28)	12 (26.09) 28 (60.87) 6 (13.04)	0.876
IMT (mm) *right (average)* *left (average)*	0.54 (0.52; 0.57) 0.55 (0.52; 0.60)	0.54 (0.50; 0.57) 0.54 (0.50; 0.59)	0.813 0.576
Cholesterol (mmol/L)	4.80 (4.23; 5.04)	4.56 (4.08; 4.95)	0.812
LDL (mmol/L)	2.73 (2.50; 3.22)	2.71 (2.39; 3.02)	0.921
HDL (mmol/L)	1.52 (1.33; 1.73)	1.34 (1.16; 1.48)	0.004
HDL (%)	34.00 (29.00; 39.00)	29.00 (25.00; 33.00)	0.047
TG (mmol/L)	0.83 (0.67; 1.08)	1.04 (0.77; 1.23)	0.063
Lp(a) (nmol/L)	10.00 (5.00; 15.00)	15.00 (7.00; 23.00)	0.579
Apo A (g/L)	1.60 (1.48; 1.72)	1.49 (1.42; 1.63)	0.071
Apo B (g/L)	0.88 (0.76; 0.94)	0.87 (0.77; 0.95)	0.850
hsCRP (mg/dl)	0.99 (0.64; 1.24)	1.15 (0.88; 1.40)	0.278
FG (mmol/L)	4.90 (4.70; 5.10)	4.95 (4.80; 5.10)	0.446
HBA1C (%)	5.00 (4.90; 5.10)	5.00 (4.90; 5.10)	0.876
LDH (U/L)	159.00 (153.00; 170.00)	168.50 (160.00; 173.00)	0.177
Bilirubin (µmol/L)	8.40 (6.20; 10.80)	9.60 (6.90; 11.40)	0.364
ALAT (U/L)	16.00 (14.00; 19.00)	20.00 (15.00; 25.00)	0.347
ASPAT (U/L)	18.00 (17.00; 19.00)	19.00 (17.00; 20.00)	0.550
GGT (U/L)	16.00 (13.00; 19.00)	24.50 (15.00; 31.00)	0.035
Fibrinogen (mg/dl)	249.00 (221.00; 294.00)	261.00 (242.00; 282.00)	0.496
ALP (U/L)	57.00 (55.00; 64.00)	59.00 (55.00; 66.00)	0.675
25(OH)D (ng/mL)	45.00 (39.00; 47.00) 43.40 ± 8.63	7.00 (7.00; 8.00) 7.13 ± 2.16	<0,001
Total calcium (mmol/L)	2.42 (2.38; 2.46)	2.40 (2.35; 2.42)	0.169
Phosphorus (mmol/L)	1.08 (1.00; 1.16)	1.06 (1.00; 1.10)	0.297

Abbreviations: BMI—body mass index; FH of DM2—family history of type 2 diabetes; FH of P-CAD—family history of premature coronary artery disease; WC—waist circumference; WHR—waist-hip ratio; VAI—visceral adiposity index; SBP—systolic blood pressure; DBP—diastolic blood pressure; IMT—intima-media thickness; LDL—low-density lipoprotein; HDL—high-density lipoprotein; TG—triglycerides; Lp(a)—lipoprotein a; Apo A—apolipoprotein A; Apo B—apolipoprotein B; hsCRP—high sensitivity C-reactive protein; FG—fasting glucose; HBA1C—glycosylated hemoglobin; LDH—lactate dehydrogenase; ALAT—alanine transaminase; ASPAT—aspartate aminotransferase; GGT—glutamyl transpeptidase; ALP—alkaline phosphatase; 25(OH)D—25 hydroxyvitamin D.

**Table 2:** Characteristics and analysis of the variants detected.

	Variant Description	
**Variant (GRCh38)**	**NC_000011.10:g.14878291G>A**	**NC_000011.10:g.14878336T>G**
**NM_024514.5**	c.1337G>A (p.Arg446Lys)	c.1331-39T>G
**Variant type**	Missense variant	Intron variant
	**Bioinformatic Analysis**	
**SIFT score**	0.0 (deleterious)	-
**PolyPhen score**	1.0 (probably damaging)	-
**SpliceAI DS (bp)**		
***acceptor loss***	0.25 (6 bp)	0.04 (−39 bp)
***donor loss***	0.00 (−36 bp)	0.00 (2 bp)
***acceptor gain***	0.00 (−31 bp)	0.00 (4 bp)
***donor gain***	0.00 (47 bp)	0.00 (−2 bp)

Abbreviations: bp—base pair; GRCh38—Genome Reference Consortium Human Build 38; SIFT—Scale Invariant Feature Transform; SpliceAI DS—SpliceAI delta score; PolyPhen—Polymorphism Phenotyping v2.

## Data Availability

The data used to support the findings of this research are available upon request.

## Supplementary Materials

The following are available online at https://www.mdpi.com/article/10.3390/biom11121867/s1, Table S1: Primer sequences for the cytochrome P450 family 2 subfamily R member 1 (*CYP2R1*) gene. Table S2: Reaction conditions for PCR using *CYP2R1*1F*, *CYP2R1*1R* and *CYP2R1*3F*, *CYP2R1*3R* primer pairs. Table S3: Reaction conditions for PCR using *CYP2R1*2F, CYP2R1*2R* primer pair. Table S4: Reaction conditions for enzymatic purification of PCR products. Table S5: Composition of the cycle sequencing reaction mixture. Table S6: Composition of the reaction mixture for the purification of sequential PCR products.

## References

[1] Sirajudeen S., Shah I., Al Menhali A. (2019). A Narrative Role of Vitamin D and Its Receptor: With Current Evidence on the Gastric Tissues. Int. J. Mol. Sci..

[2] Jäpelt R.B., Jakobsen J. (2013). Vitamin D in plants: A review of occurrence, analysis, and biosynthesis. Front. Plant Sci..

[3] Nair R., Maseeh A. (2012). Vitamin D: The sunshine vitamin. J. Pharmacol. Pharmacother..

[4] Sahota O. (2014). Understanding vitamin D deficiency. Age Ageing.

[5] Dziedzic E.A., Gąsior J.S., Pawłowski M., Wodejko-Kucharska B., Saniewski T., Marcisz A., Dąbrowski M.J. (2019). Vitamin D level is associated with severity of coronary artery atherosclerosis and incidence of acute coronary syndromes in non-diabetic cardiac patients. Arch. Med. Sci..

[6] Mozos I., Marginean O. (2015). Links between Vitamin D Deficiency and Cardiovascular Diseases. BioMed Res. Int..

[7] Judd S.E., Tangpricha V. (2009). Vitamin D Deficiency and Risk for Cardiovascular Disease. Am. J. Med. Sci..

[8] Płudowski P., Karczmarewicz E., Bayer M., Carter G., Chlebna-Sokół D., Czech-Kowalska J., Dębski R., Decsi T., Dobrzanska A., Franek E. (2013). Practical guidelines for the supplementation of vitamin D and the treatment of deficits in Central Europe—Recommended vitamin D intakes in the general population and groups at risk of vitamin D deficiency. Endokrynol. Pol..

[9] Rusińska A., Pludowski P., Walczak M., Borszewska-Kornacka M.K., Bossowski A., Chlebna-Sokół D., Czech-Kowalska J., Dobrzanska A., Franek E., Helwich E. (2018). Vitamin D Supplementation Guidelines for General Population and Groups at Risk of Vitamin D Deficiency in Poland—Recommendations of the Polish Society of Pediatric Endocrinology and Diabetes and the Expert Panel With Participation of National Specialist Consultants and Representatives of Scientific Societies—2018 Update. Front. Endocrinol..

[10] Bikle D. (2017). Vitamin D. Production, Metabolism, and Mechanisms of Action.

[11] HUGO Gene Nomenclature Committee CNR2. https://www.genenames.org/data/gene-symbol-report/#!/hgnc_id/HGNC:2160.

[12] Thacher T.D., Fischer P.R., Singh R.J., Roizen J., Levine M.A. (2015). CYP2R1Mutations Impair Generation of 25-hydroxyvitamin D and Cause an Atypical Form of Vitamin D Deficiency. J. Clin. Endocrinol. Metab..

[13] Strushkevich N., Usanov S.A., Plotnikov A.N., Jones G., Park H.-W. (2008). Structural Analysis of CYP2R1 in Complex with Vitamin D3. J. Mol. Biol..

[14] Fetahu I.S., Höbaus J., Kállay E. (2014). Vitamin D and the epigenome. Front. Physiol..

[15] Kämpe A., Enlund-Cerullo M., Valkama S., Holmlund-Suila E., Rosendahl J., Hauta-Alus H., Pekkinen M., Andersson S., Mäkitie O. (2019). Genetic variation in GC and CYP2R1 affects 25-hydroxyvitamin D concentration and skeletal parameters: A genome-wide association study in 24-month-old Finnish children. PLoS Genet..

[16] O’Brien K.M., Sandler D.P., Shi M., Harmon Q.E., Taylor J., Weinberg C. (2018). Genome-Wide Association Study of Serum 25-Hydroxyvitamin D in US Women. Front. Genet..

[17] Cheng J.B., Levine M., Bell N.H., Mangelsdorf D., Russell D. (2004). Genetic evidence that the human CYP2R1 enzyme is a key vitamin D 25-hydroxylase. Proc. Natl. Acad. Sci. USA.

[18] CYP2R1 Cytochrome P450 Family 2 Subfamily R Member 1 Homo Sapiens (Human)-Gene-NCBI. https://www.ncbi.nlm.nih.gov/gene?Db=gene&Cmd=DetailsSearch&Term=120227.

[19] Roizen J.D., Levine M.A., Martin H., Roger B., Edward G., David G. (2017). The role of genetic variation in CYP2R1, the principal vitamin D 25-hydroxylase, in vitamin D homeostasis. Vitamin D: Volume 2: Health, Disease and Therapeutics.

[20] Osadnik T., Osadnik K., Pawlas N., Strzelczyk J., Kasperczyk J., Poloński L., Gąsior M. (2019). Metabolic and genetic profiling of young adults with and without a family history of premature coronary heart disease (MAGNETIC). Study design and methodology. Arch. Med. Sci..

[21] Rausch T., Fritz M.H.-Y., Untergasser A., Benes V. (2020). Tracy: Basecalling, alignment, assembly and deconvolution of sanger chromatogram trace files. BMC Genom..

[22] Bcftools by Samtools. https://samtools.github.io/bcftools/.

[23] McLaren W., Gil L., Hunt S.E., Riat H.S., Ritchie G.R.S., Thormann A., Flicek P., Cunningham F. (2016). The Ensembl Variant Effect Predictor. Genome Biol..

[24] GeneStudio Pro. https://genestudio-pro.software.informer.com/.

[25] Ng P.C. (2003). SIFT: Predicting amino acid changes that affect protein function. Nucleic Acids Res..

[26] Pathogenicity Predictions. https://m.ensembl.org/info/genome/variation/prediction/protein_function.html.

[27] Adzhubei I.A., Schmidt S., Peshkin L., Ramensky V.E., Gerasimova A., Bork P., Kondrashov A.S., Sunyaev S.R. (2010). A method and server for predicting damaging missense mutations. Nat. Methods.

[28] Jaganathan K., Panagiotopoulou S.K., McRae J.F., Darbandi S.F., Knowles D., Li Y.I., Kosmicki J.A., Arbelaez J., Cui W., Schwartz G.B. (2019). Predicting Splicing from Primary Sequence with Deep Learning. Cell.

[29] Kim S.-H., Yang S., Lim K.-H., Ko E., Jang H.-J., Kang M., Suh P.-G., Joo J.-Y. (2021). Prediction of Alzheimer’s disease-specific phospholipase c gamma-1 SNV by deep learning-based approach for high-throughput screening. Proc. Natl. Acad. Sci. USA.

[30] Chr11: 14.88M-14.89M-Genome Data Viewer-NCBI. https://www.ncbi.nlm.nih.gov/genome/gdv/browser/nucleotide/?id=NM_024514.5.

[31] PDBe-KB Protein Pages. https://www.ebi.ac.uk/pdbe/pdbe-kb/proteins/Q6VVX0.

[32] Amino Acids-Arginine R (Arg). http://www.biology.arizona.edu/biochemistry/problem_sets/aa/Arginine.html.

[33] Berg J.M., Tymoczko J.L., Stryer L. (2002). Amino Acids Are Encoded by Groups of Three Bases Starting from a Fixed Point.

[34] Schneider F. (1978). Die Funktion des Arginins in den Enzymen. Naturwissenschaften.

[35] Amino Acids–Lysine. http://www.biology.arizona.edu/biochemistry/problem_sets/aa/Lysine.html.

[36] Li L., Vorobyov I., Allen T.W. (2013). The Different Interactions of Lysine and Arginine Side Chains with Lipid Membranes. J. Phys. Chem. B.

[37] Sokalingam S., Raghunathan G., Soundrarajan N., Lee S.-G. (2012). A Study on the Effect of Surface Lysine to Arginine Mutagenesis on Protein Stability and Structure Using Green Fluorescent Protein. PLoS ONE.

[38] Musafia B., Buchner V., Arad D. (1995). Complex Salt Bridges in Proteins: Statistical Analysis of Structure and Function. J. Mol. Biol..

[39] Matsutani M., Hirakawa H., Nishikura M., Soemphol W., Ali I.A.I., Yakushi T., Matsushita K. (2011). Increased number of Arginine-based salt bridges contributes to the thermotolerance of thermotolerant acetic acid bacteria, Acetobacter tropicalis SKU1100. Biochem. Biophys. Res. Commun..

[40] Kumar S., Nussinov R. (1999). Salt bridge stability in monomeric proteins. J. Mol. Biol..

[41] Strub C., Alies C., Lougarre A., Ladurantie C., Czaplicki J., Fournier D. (2004). Mutation of exposed hydrophobic amino acids to arginine to increase protein stability. BMC Biochem..

[42] Chan C.-H., Yu T.-H., Wong K.-B. (2011). Stabilizing Salt-Bridge Enhances Protein Thermostability by Reducing the Heat Capacity Change of Unfolding. PLoS ONE.

[43] Dong A.N., Tan B.H., Pan Y., Ong C.E. (2021). The CYP2R1 Enzyme: Structure, Function, Enzymatic Properties and Genetic Polymorphism. J. Pharm. Pharm. Sci..

[44] Al Mutair A.N., Nasrat G.H., Russell D. (2012). Mutation of the CYP2R1 Vitamin D 25-Hydroxylase in a Saudi Arabian Family with Severe Vitamin D Deficiency. J. Clin. Endocrinol. Metab..

[45] Molin A., Wiedemann A., Demers N., Kaufmann M., Cao J.D., Mainard L., Dousset B., Journeau P., Abeguile G., Coudray N. (2017). Vitamin D-Dependent Rickets Type 1B (25-Hydroxylase Deficiency): A Rare Condition or a Misdiagnosed Condition?. J. Bone Miner. Res..

[46] Ramos-Lopez E., Brück P., Jansen T., Herwig J., Badenhoop K. (2007). CYP2R1 (vitamin D 25-hydroxylase) gene is associated with susceptibility to type 1 diabetes and vitamin D levels in Germans. Diabetes/Metab. Res. Rev..

[47] Duan L., Xue Z., Ji H., Zhang D., Wang Y. (2018). Effects of CYP2R1 gene variants on vitamin D levels and status: A systematic review and meta-analysis. Gene.

[48] Lafi Z.M., Irshaid Y.M., El-Khateeb M., Ajlouni K.M., Hyassat D. (2015). Association of rs7041 and rs4588 Polymorphisms of the Vitamin D Binding Protein and the rs10741657 Polymorphism of CYP2R1 with Vitamin D Status Among Jordanian Patients. Genet. Test. Mol. Biomark..

[49] Slater N.A., Rager M.L., Havrda D.E., Harralson A.F. (2017). Genetic Variation in CYP2R1 and GC Genes Associated With Vitamin D Deficiency Status. J. Pharm. Pr..

[50] Nissen J., Rasmussen L.B., Ravn-Haren G., Andersen E.W., Hansen B., Andersen R., Mejborn H., Madsen K.H., Vogel U. (2014). Common Variants in CYP2R1 and GC Genes Predict Vitamin D Concentrations in Healthy Danish Children and Adults. PLoS ONE.

[51] Hassanein S.I., Abu El Maaty M.A., Sleem H.M., Gad M.Z. (2014). Triangular relationship between single nucleotide polymorphisms in the CYP2R1 gene (rs10741657 and rs12794714), 25-hydroxyvitamin d levels, and coronary artery disease incidence. Biomarkers.

[52] Saponaro F., Saba A., Zucchi R. (2020). An Update on Vitamin D Metabolism. Int. J. Mol. Sci..

[53] Morris H.A. (2014). Vitamin D Metabolism and Molecular Modes of Action: New Insights into Vitamin D Activities. Med. Res. J..

[54] Jones G., Prosser D.E., Kaufmann M. (2014). Cytochrome P450-mediated metabolism of vitamin D. J. Lipid Res..

[55] Zhu J., DeLuca H.F. (2012). Vitamin D 25-hydroxylase–Four decades of searching, are we there yet?. Arch. Biochem. Biophys..

[56] Gupta R.P., He Y.A., Patrick K.S., Halpert J.R., Bell N.H. (2005). CYP3A4 Is a Vitamin D-24- and 25-Hydroxylase: Analysis of Structure Function by Site-Directed Mutagenesis. J. Clin. Endocrinol. Metab..

[57] Aiba I., Yamasaki T., Shinki T., Izumi S., Yamamoto K., Yamada S., Terato H., Ide H., Ohyama Y. (2006). Characterization of rat and human CYP2J enzymes as Vitamin D 25-hydroxylases. Steroids.

[58] Bergadà L., Pallares J., Arcidiacono M.V., Cardus A., Santacana M., Valls J., Cao G., Fernàndez E., Dolcet X., Dusso A.S. (2014). Role of local bioactivation of vitamin D by CYP27A1 and CYP2R1 in the control of cell growth in normal endometrium and endometrial carcinoma. Lab. Investig..

[59] Zhu J.G., Ochalek J.T., Kaufmann M., Jones G., DeLuca H.F. (2013). CYP2R1 is a major, but not exclusive, contributor to 25-hydroxyvitamin D production in vivo. Proc. Natl. Acad. Sci. USA.

[60] Putscher E., Hecker M., Fitzner B., Lorenz P., Zettl U. (2021). Principles and Practical Considerations for the Analysis of Disease-Associated Alternative Splicing Events Using the Gateway Cloning-Based Minigene Vectors pDESTsplice and pSpliceExpress. Int. J. Mol. Sci..

[61] Sanz D.J., Hollywood J.A., Scallan M.F., Harrison P.T. (2017). Cas9/gRNA targeted excision of cystic fibrosis-causing deep-intronic splicing mutations restores normal splicing of CFTR mRNA. PLoS ONE.

