# Supplementary material for: New Variants of the Cytochrome P450 2R1 (CYP2R1) Gene in Individuals with Severe Vitamin D-Activating Enzyme 25(OH)D Deficiency
Source: Biomolecules. 2021 Dec 12;11(12):1867. doi: 10.3390/biom11121867 (PMC8699237; doi:10.3390/biom11121867)
Supplement: Supplementary file 1 [file biomolecules-11-01867-s001.zip › biomolecules-1477803-supplementary.pdf]

New Variants of the Cytochrome P450 2R1 (*CYP2R1)* Gene in Individuals with Severe Vitamin D-activating Enzyme 25(OH)D Deficiency

Martyna Fronczek[1,2], Joanna Katarzyna Strzelczyk[1], Krzysztof Biernacki[1], Silvia Salatino[3], Tadeusz Osadnik[2], Zofia Ostrowska[1]

Table S1. Primer sequences for the cytochrome P450 family 2 subfamily R member 1 *(CYP2R1)* gene.

Primer Name	Primer Sequence [5'→ 3']	Chromosome Position	Amplicon Length (bp)
*CYP2R1*1F*	5'-CTGTGTTCATTTGGCTTTTTGATGC-3'	Ch11: 14878000 - 14878499	500
*CYP2R1*1R*	5'-ACAAAAGGAGGTAAATGAATGGGT-3'		
*CYP2R1*2F*	5'-GTGCATGGCCAAGACTCAAAAAC-3'	Ch11: 14880058 - 14880883	826
*CYP2R1*2R*	5'-TAGGAGGACAATTTGGAGAAGGAT-3'		
*CYP2R1*3F*	5'-GTCCTTTACACAAACCATGCAAC-3'	Ch11: 14885321 - 14886113	814
*CYP2R1*3R*	5'-GTGACTTTAGGCACTGAATGGC-3'		

Table S2. Reaction conditions for PCR using *CYP2R1*1F, CYP2R1*1R* and *CYP2R1*3F, CYP2R1*3R* primer pairs.

PCR Conditions						
**Reagent**	**Volume**	**Thermocycling Conditions**	**Temperature [°C]**	**Time [mm:ss]**	**Cycle**	**Instrument**
DNA	300 ng per reaction	Initial denaturation	94	03:00	1	SimpliAmp™ ThermalCycler (Thermo Fisher, USA)
10X Optimized DyNAzyme Buffer with 1.5 mM $MgCl_2$	2,5 μL	Denaturation	94	00:45	x 30	
10 mM dNTPs	0,2 μL	Annealing	58	00:30		
DyNAzyme II DNA Polymerase 2 U/μL	0,6 μL	Extension	72	00:45		
10 μM reverse primer	1 μL	Final extension	72	07:00	1	
10 μM forward primer	1 μL	Storage	4	∞		
RNase-free and DNase-free water	up to final volume					
Final reaction volume	25 μL					

Table S3. Reaction conditions for PCR using *CYP2R1*2F, CYP2R1*2R* primer pair.

PCR Conditions						
**Reagent**	**Volume**	**Thermocycling conditions**	**Temperature [°C]**	**Time [mm:ss]**	**Cycle**	**Instrument**
DNA	300 ng per reaction	Initial denaturation	94	03:00	1	SimpliAmp™ ThermalCycler (Thermo Fisher, USA)
10X Optimized DyNAzyme Buffer with 1.5 mM $MgCl_2$	2,5 μL	Denaturation	94	00:45	x 30	
10 mM dNTPs	0,2 μL	Annealing	60	00:30		
DyNAzyme II DNA Polymerase 2 U/μL	0,6 μL	Extension	72	00:45		
10μM reverse primer	1 μL	Final extension	72	07:00	1	
10 μM forward primer	1 μL	Storage	4	∞		
RNase-free and DNase-free water	up to final volume					
Final reaction volume	25 μL					

Table S4. Reaction conditions for enzymatic purification of PCR products.

Reaction Conditions					
**Reagent**	**Volume**	**Thermocycling conditions**	**Temperature [°C]**	**Time [mm:ss]**	**Instrument**
Exo-BAP Mix	2 µL	Enzyme activation	37	15:00	SimpliAmp™ ThermalCycler (Thermo Fisher, USA)
PCR product	5 µL	Enzyme inactivation	80	15:00	
Final reaction volume	25 µL	Storage	4	∞	

Table S5. Composition of the cycle sequencing reaction mixture.

PCR Reaction Composition		PCR Thermal Conditions				
**Reagent**	**Volume [µL]**	**Thermocycling conditions**	**Temperature [°C]**	**Time [mm:ss]**	**Cycle**	**Instrument**
BigDye™ Terminator 3.1 Ready Reaction Mix	4	Initial denaturation	96	01:00	1	SimpliAmp™ ThermalCycler (Thermo Fisher, USA)
BigDye™ Terminator v1.1 & v3.1 5X Sequencing Buffer	2	Denaturation	96	00:10	x 25 Ramp rate 1°C/s	
Forward/Reverse primer (3.2 µM)	1	Annealing	50	00:05		
Purified PCR product	10 ng per reaction	Extension	60	04:00		
RNase-free and DNase-free water	up to final volume	Storage	4	∞		
Final reaction volume	20					

Table S6. Composition of the reaction mixture for the purification of sequential PCR products.

Reaction Mixture		Conditions	
**Reagent**	**Volume [µL]**	**Shaking conditions**	**Instrument**
Purified sequencing PCR product	20	2000 rpm	IKA MS3
BigDye® XTerminator™ Solution	20		
SAM™ Solution	90		
